# Índice Hemodinâmico Agudo Prediz Mortalidade Hospitalar em Insuficiência Cardíaca Descompensada

**DOI:** 10.36660/abc.20201294

**Published:** 2021-01-27

**Authors:** Sofia Alegria

**Affiliations:** 1 Hospital Garcia de Orta EPE Almada Portugal Hospital Garcia de Orta EPE – Cardiologia, Almada - Portugal

**Keywords:** Insuficiência Cardíaca, Fibrilação Atrial, Volume Sistólico, Hemodinâmica, Baixo Débito Cardíaco, Frequência Cardíaca

Embora a insuficiência cardíaca aguda (ICA) esteja associada a mortalidade hospitalar significativa (em torno de 9-11% de acordo com a taxa de mortalidade no registro BREATHE) e altas taxas de reinternação após a alta, as opções para o manejo desses pacientes permanecem limitadas.^1^

Uma vez que a sobrevida global é determinada principalmente pelo manejo inicial, uma estratificação de risco individual precisa e precoce pode ajudar os médicos a escolher a intensidade do cuidado necessário e promover a tomada de decisões médicas feita sob medida, com melhora do prognóstico.^2^

O artigo de Castro et al.^3^ fornece uma ferramenta simples, a ser utilizada à beira do leito, para estratificar a população de pacientes com ICA com fração de ejeção reduzida, com base no cálculo do índice hemodinâmico agudo (IHA) (IHA= $\left(IHA=\frac{\text{ pressao de pulso }x\text{ frequencia cardiaca }}{1000}\right)$ )que morreram, o que alguns estudos sugerem estar associado a maior mortalidade, especialmente em pacientes com IC e fibrilação atrial.^7^

O achado de que a PAS baixa estava associada à mortalidade também é consistente com outros estudos que demonstraram a importância prognóstica desse parâmetro, provavelmente porque PAS baixa e pressão de pulso estreita proporcional são marcadores de hipoperfusão.^7^ O registro OPTIMIZE-HF^4^ mostrou que valores de PAS abaixo de 120 mmHg caracterizavam os pacientes com ICA que apresentavam prognóstico desfavorável apesar da terapia medicamentosa, mas no estudo atual, a pressão arterial abaixo de 120 mmHg não foi independentemente relacionada à mortalidade em uma análise multivariada.

Hipotetizaram que a PAS elevada na admissão observada na maioria dos pacientes com ICA pode estar relacionada à ativação neuro-hormonal e de citocinas resultando em pós-carga aumentada, mas a fisiopatologia pode diferir em pacientes que apresentam PAS baixa e, consequentemente, pressão de pulso baixa, o que mais provavelmente pode significar doença em estágio avançado ou terminal com baixo débito cardíaco e sinais de hipoperfusão de órgãos. Também é razoável supor que pacientes com uma PAS elevada podem responder mais favoravelmente a vasodilatadores e antagonistas neuro-hormonais. No entanto, nenhum dos agentes farmacológicos estudados em ensaios recentes (vasodilatadores, inodilatadores e sensibilizadores de cálcio) melhorou os resultados clínicos.^5 , 8^

Além disso, a maioria das estimativas de risco foram derivadas de conjuntos de dados de ensaios clínicos, que podem não ser representativos de grandes populações de pacientes internados por IC.^1^ Além disso, o número de variáveis e funções matemáticas envolvidas frequentemente requerem acesso a um computador ou calculadora eletrônica para gerar um escore e determinar o risco, tornando-os impraticáveis para avaliação à beira do leito, e dependem da medição de biomarcadores, treinamento da equipe médica e tecnologia que pode não estar amplamente disponível.^4 , 9^ Em contraste, as medidas de FC e PA estão disponíveis em praticamente todos os locais de serviços de saúde com boa precisão e requerem treinamento mínimo, o que torna o IHA um marcador prognóstico prático, objetivo e de fácil obtenção.

Algumas limitações deste estudo devem ser reconhecidas. Este foi um estudo observacional incluindo menos de 500 pacientes, potencialmente não representativo de toda a população de pacientes com ICA e seus achados devem ser considerados como geradores de hipóteses e posteriormente validados em estudos prospectivos em outras populações.

Os resultados de estudos baseados em registros, como o Registro BREATHE, podem ajudar adicionalmente a definir modelos úteis para o desenho de ensaios clínicos para avaliar terapias para IC, uma vez que permitem que o risco seja equilibrado entre os grupos de tratamento e permitem critérios de inclusão seletivos para incluir apenas pacientes com alto risco de mortalidade intra-hospitalar. Eles também contribuem para o desenvolvimento de um modelo de predição de risco clínico para ICA, permitindo que os médicos estejam mais bem equipados para otimizar a utilização de recursos hospitalares com base em estimativas de risco específicas do paciente e, adicionalmente, as decisões terapêuticas podem eventualmente ser guiadas por estimativas de risco. Pacientes com estimativa de menor risco podem ser tratados com monitoramento menos intensivo e terapias disponíveis em uma unidade de telemetria ou enfermaria de hospital, enquanto um paciente com maior risco estimado pode necessitar de tratamento mais intensivo em uma unidade de terapia intensiva ou coronariana.^2^ Entretanto, devemos ter em mente que esses modelos aprimoram, mas não substituem, a avaliação do médico.
