# Supplementary material for: Índice Hemodinâmico Agudo Prediz Mortalidade Hospitalar em Insuficiência Cardíaca Descompensada
Source: Arq Bras Cardiol. 2021 Jan 27;116(1):87–8. [Article in Portuguese] doi: 10.36660/abc.20201294 (PMC8159497; doi:10.36660/abc.20201294)
Supplement: Translation

# Translation: Acute Hemodynamic Index Predicts In-Hospital Mortality in Acute Decompensated Heart Failure

**Authors:** Sofia Alegria

**Affiliations:** 1 Portugal Hospital Garcia de Orta EPE – Cardiologia, Almada - Portugal

**Keywords:** Heart Failure, Atrial Fibrillation, Stroke Volume, Hemodynamic, Cardiac Output Low, Heart Rate

Although acute heart failure (AHF) is associated with significant in-hospital mortality (around 9-11% in concordance with the mortality rate in the BREATHE registry) and high rates of rehospitalization after discharge, options for the management of these patients remain limited.^1^

Since overall survival is mainly determined by the initial management, accurate and early individual risk stratification can help physicians choose the intensity of care required and promote tailored medical decision-making with improvement of prognosis.^2^

The manuscript by Castro et al.^3^provides a simple, bedside tool, to stratify the population of patients with AHF with reduced ejection fraction, based on the calculation of the acute hemodynamic index (AHI) (AHI= $\left(AHI=\frac{\text{ pulse pressure }x\text{ heart rate }}{1000}\right)$ ) at admission. The authors report that patients with low AHI (≤ 4 mmHg bpm) had an in-hospital mortality that was 2.5 times higher than patients with an higher AHI.

In the present analysis from the BREATHE registry only patients with evidence of left ventricle ejection fraction below 40% were included, contrary to most of the previous publications. Although previous studies, generally based on outpatients with chronic heart failure (HF), have identified a number of variables that are associated with increased mortality, including etiology, patient age, peak oxygen consumption, left ventricular ejection fraction, serum sodium concentration, and B-type natriuretic peptide concentration, several factors have limited the development of similar models in patients with AHF, such as lack of a consistent definition of AHF, incomplete data in administrative data sets, and varying statistical methods. Consequently, unlike acute coronary syndromes, in which several systems have been developed for risk stratification, no clinically practical method of risk stratification exists for patients with AHF.^4^

Results from the American multicenter ADHERE HF Registry identified blood urea nitrogen level, systolic blood pressure (SBP), heart rate (HR), and age as the most significant predictors of mortality in patients with AHF.^1^Others studies have also shown that an increased HR predicts prognosis in patients presenting with HF.^5^Autonomic imbalance resulting from sympathetic overactivity and parasympathetic withdrawal is likely to be the underlying mechanism of increased HR in HF. Several pathophysiologic mechanisms, including increased myocardial oxygen consumption, reduced diastolic filling times, compromised coronary perfusion with induction of myocardial ischemia, and precipitation of rhythm disturbances have been proposed to explain the association between higher HR and worse outcomes.^2^However, it has also been demonstrated that chronotropic incompetence, especially in patients with chronic HF, is associated with reduced functional capacity and poor survival.^6^In the present study an higher HR was not associated with worse outcomes. In fact, patients who died had a mean HR of 82 bpm at admission while those who survived had 90 bpm. Nevertheless, in the multivariate analysis HR was not an independent predictor of mortality. The association between a lower HR and mortality was unexpected and we can speculate that this might be due to the higher prevalence of treatment with digitalis in patients who died, which some studies suggest to be associated with higher mortality, especially in patients with HF and atrial fibrillation.^7^

The finding that low SBP was associated with mortality is also consistent with other studies that have demonstrated the prognostic importance of this parameter, probably because low SBP and narrow proportional pulse pressure are markers of hypoperfusion.^7^The OPTIMIZE-HF^4^registry found that SBP values below 120 mmHg characterized patients with AHF who had poor prognosis despite medical therapy, but in the current study, blood pressure below 120 mmHg was not independently related to mortality in a multivariate analysis. It has been hypothesized that the elevated SBP at admission observed in the majority of AHF patients may be related to neurohormonal and cytokine activation resulting in increased afterload, but the pathophysiology may differ in patients presenting with low SBP and consequently low pulse pressure, who may be more likely to have advanced or end-stage disease with low cardiac output and signs of organ hypoperfusion. It is also reasonable to hypothesize that patients with an elevated SBP may respond more favorably to vasodilators and neurohormonal antagonists. Nevertheless, none of the pharmacologic agents studied in recent trials (vasodilators, inodilators, and calcium sensitizers) has improved clinical outcomes.^5 , 8^

In addition, most risk estimates have been derived from clinical trial datasets, which may not be representative of broad populations of patients admitted for HF.^1^Also, the number of variables and mathematical functions involved frequently require access to a computer or an electronic calculator to generate a score and to determine risk, making them impractical for bedside assessment, and rely on biomarker measurement, medical staff training, and technology that may not be widely available.^4 , 9^In contrast, HR and BP measurements are available in virtually any healthcare facility with good accuracy and requiring minimal training, which makes AHI a practical, objective, and easily obtained prognostic marker.

Some limitations of this study should be acknowledged. It was an observational study including less than 500 patients, potentially not representative of the whole population of patients with AHF and its findings should be considered hypothesis-generating and subsequently validated in prospective studies in other populations.

The results of registry-based studies, like the BREATHE Registry, may additionally help to define models useful for the design of clinical trials to evaluate HF therapies, since they permit risk to be balanced across treatment groups and allow for selective inclusion criteria in order to enroll only patients at high risk for in-hospital mortality. They also contribute to the development of a clinical risk prediction model for AHF allowing clinicians to be better equipped to optimize in-hospital resource utilization based on patient-specific risk estimates, and additionally therapeutic decisions may eventually be guided by risk estimates as well. Patients estimated to be at a lower risk can be managed with less intensive monitoring and therapies available on a telemetry unit or hospital ward, whereas a patient estimated to be at a higher risk may require more intensive management in an intensive or coronary care unit.^2^Nevertheless, we should bear in mind that these models enhance, but don’t replace, physician assessment.
